# Metastatic Calcinosis Cutis: A Case in a Child with Acute Pre-B Cell Lymphoblastic Leukemia

**DOI:** 10.1155/2015/384821

**Published:** 2015-08-05

**Authors:** Juan Pablo Castanedo-Cázares, Amalia Reyes-Herrera, Diana Hernández-Blanco, Cuauhtémoc Oros-Ovalle, Bertha Torres-Álvarez

**Affiliations:** ^1^Department of Dermatology, Hospital Central Dr. Ignacio Morones Prieto, Universidad Autónoma de San Luis Potosí, 78210 San Luis Potosí, SLP, Mexico; ^2^Department of Pathology, Hospital Central Dr. Ignacio Morones Prieto, Universidad Autónoma de San Luis Potosí, 78210 San Luis Potosí, SLP, Mexico

## Abstract

Hypercalcemia in children with malignancy is an uncommon condition. It has been described in leukemia patients with impaired renal excretion of calcium or osteolytic lesions. Metastatic calcinosis cutis (MCC) may develop if hypercalcemia persists. We report the case of a 5-year-old girl with an atypical dermatosis and unspecific gastrointestinal symptoms. Considered clinical diagnoses were xanthomas, histiocytosis, molluscum contagiosum, and nongenital warts. Cutaneous histological analysis showed amorphous basophilic deposits in the dermis suggestive of calcium deposits. Laboratory tests confirmed serum hypercalcemia. Extensive investigations such as bone marrow biopsy established the diagnosis of an acute pre-B cell lymphoblastic leukemia. Hypercalcemia in hematopoietic malignancies is unusual, especially as initial manifestation of the disease. Careful review of the literature fails to reveal previous reports of these peculiar cutaneous lesions of MCC in children with leukemia.

## 1. Introduction

Hypercalcemia usually results in nonspecific constitutional and gastrointestinal symptoms, such as nausea, vomiting, anorexia, and weight loss. Its presence in children with malignancy is very rare but has been linked to rhabdomyosarcoma, hepatoblastoma, Hodgkin's and non-Hodgkin's lymphoma, brain tumors, neuroblastoma, angiosarcoma, and acute lymphoblastic and myeloid acute leukemia [1]. Metastatic calcinosis cutis (MCC) occurs in undamaged tissues and is associated with elevated serum phosphate and/or calcium levels, most frequently in adults with end stage renal disease. It is an uncommon finding in pediatric leukemia patients [2, 3]. This case report highlights the importance of skin features in the diagnosis of a hematological disease in a child with nonspecific symptoms.

## 2. Case Presentation

A 5-year-old girl with a history of nausea, vomiting, abdominal pain, and diarrhea, who had been hospitalized without a definite diagnosis of her illness on multiple occasions before her consultation, was admitted because of diarrhea and moderate dehydration.

**Figure 1:**
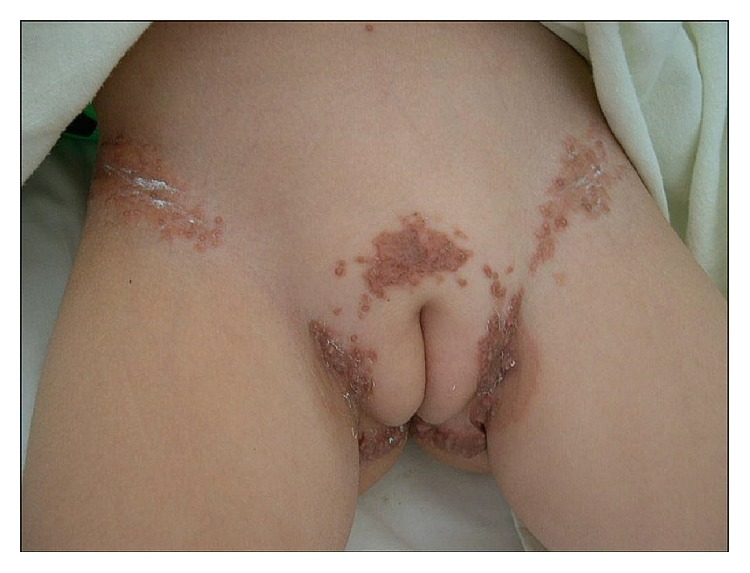
Plaques of confluent papules, reddish brown on the groins, pubis, and gluteal fold, firm, and well demarcated.

**Figure 2:**
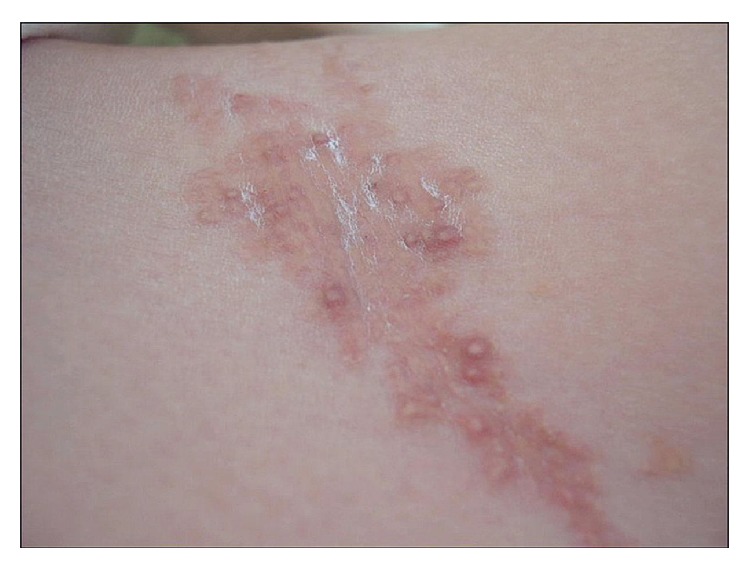
Multiple pinkish, pearly, flesh colored papules.

**Figure 3:**
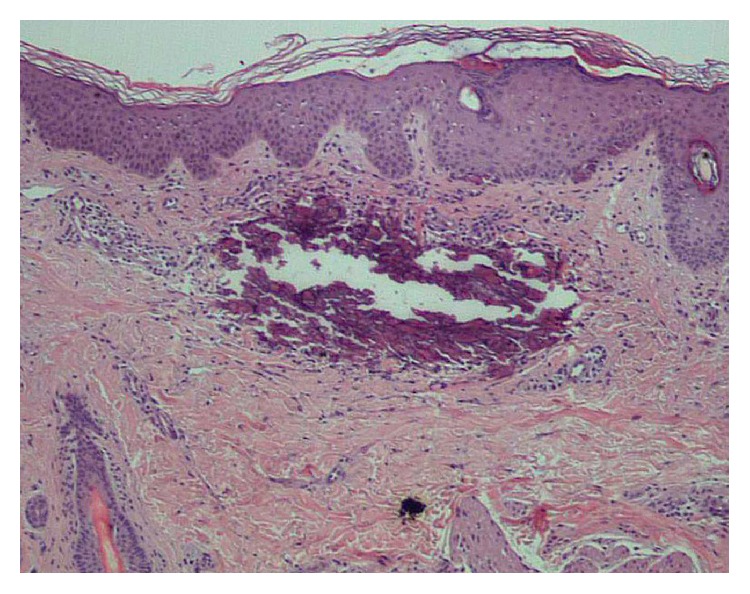
Hematoxylin and eosin stain reveals amorphous, basophilic deposit in reticular dermis of calcium deposits; original magnification ×100.

Physical examination revealed asymptomatic large plaques of confluent papules, reddish brown on the groins, pubis, and gluteus fold, firm, and well demarcated, approximately 2 × 7 cm in size of four-week duration (Figures 1 and 2). Neither hepatosplenomegaly nor palpable lymphadenopathies were detected. The clinical diagnosis of skin lesions was xanthomas, histiocytosis, molluscum contagiosum, or nongenital warts. Laboratory tests on admission were normal, including hemogram with hemoglobin level of 11.5 g/dL, a white cell differential count of 70% neutrophils, and 25% lymphocytes as well as phosphorous and parathyroid hormone levels, but an abnormal high level of serum calcium of 17.9 mg/dL. A 4-mm punch skin biopsy was taken, and subsequent hematoxylin and eosin stain revealed an amorphous and basophilic deposit in reticular dermis, highly suggestive of calcium deposits (Figure 3). Von Kossa staining was positive and the diagnosis of calcinosis cutis was confirmed. Chest X-ray was normal and kidney ultrasound suggested nephrocalcinosis; however renal function parameters remained normal. There was no evidence of other visceral calcifications.

**Figure 4:**
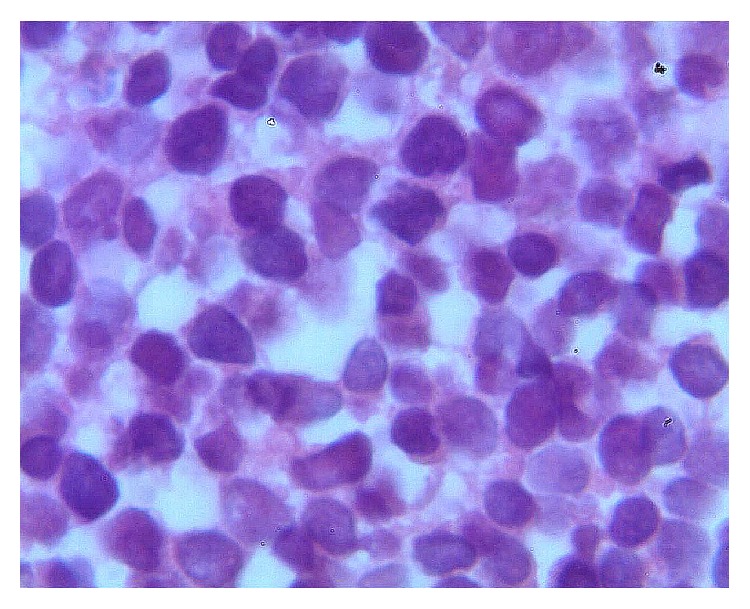
Hematoxylin and eosin stain of bone marrow biopsy showed significant hypercellularity with over 90% blasts with L1 morphology; original magnification ×1000.

**Figure 5:**
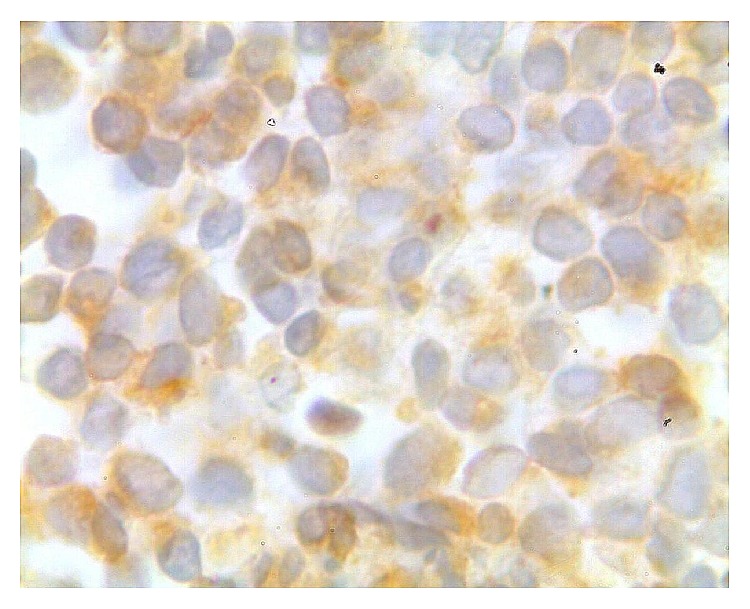
Immunohistochemical stain detected neoplastic cells positive for CD79a; original magnification ×1000.

Finally, a bone marrow biopsy revealed a significant hypercellularity with over 90% blasts with L1 morphology (Figure 4). Immunohistochemical exam detected neoplastic cells positive for CD20 and CD79a indicating an infiltrate of B cell precursors (Figure 5). Flow cytometry of bone marrow was also consistent with early pre-B cell lymphoblastic leukemia as immunolabeling was negative for surface immunoglobulin but positive for CD10, CD20, and CD19 [4]. Two weeks after admission, follow-up blood work revealed hemoglobin 9.2 g/dL with a white blood count of 2710/*μ*L, with 82% neutrophils and 15% lymphocytes and an elevated level of lactate dehydrogenase (LDH, 2440 U/L; normal < 213 U/L) was detected. The patient was treated with three cycles of vincristine, daunorubicin, and L-asparaginase. After that the level of serum calcium was 9.4 mg/dL and the level of lactate dehydrogenase was reduced to a normal level. Skin lesions did not regress but they did not expand either, as no particular treatment was offered for these lesions. One month after the last chemotherapy cycle, the patient developed central nervous system leukemia and intrathecal methotrexate was given; however there was no response for that and treatment with cranial radiotherapy was started. Four months after her diagnosis the patient presented pancytopenia and died from sepsis.

## 3. Discussion

Calcinosis cutis is classified as metastatic, dystrophic, idiopathic, or iatrogenic. Dystrophic calcinosis cutis is the most common form; it appears secondary to tissue damage in a setting of underlying disease associated with normal serum calcium and phosphate levels [3]. Idiopathic type generally occurs in childhood or adolescence and can be classified as solitary or multiple, sporadic or associated with Down syndrome [4, 5]. The iatrogenic type occurs following treatment in the setting of intravenous extravasation of calcium chloride, calcium gluconate, or phosphate [6].

Hypercalcemia is defined as total serum calcium concentration greater than 12 mg/dL, but clinical symptoms usually appear when concentration is 15 mg/dL or greater [7].

MCC occurs when there is hypercalcemia or hyperphosphatemia secondary to abnormal calcium, phosphate metabolism, or both leading to spontaneous calcium/phosphate deposits [6]. Characteristically, calcifications appear at periarticular sites, and their size and number seem to correlate with the degree of hyperphosphatemia or hypercalcemia [3]. Metastatic calcification (MC) may occur not only within the cutaneous and subcutaneous tissues, but also within other organs or tissues such as the kidneys, lungs, stomach, and blood vessels [5, 8]. MC is more common among adults, particularly in those affected by chronic renal failure. Other conditions related to its occurrence are hypervitaminosis D, the milk-alkali syndrome, Albright hereditary osteodystrophy, neoplasms associated with bony destruction (lymphomas, leukemias, multiple myeloma, and metastatic carcinomas), sarcoidosis, and pseudohyperparathyroidism [6]. Apparently there are only four cases in the literature of MC in children; two of them presented also MCC: one child with end-stage renal disease, metastatic calcinosis of lungs, and cutaneous necrosis of buttocks and legs [9] and the other case was an 18-month-old infant with massive metastatic pulmonary calcification in a leukemic monocytic leukemia [10]. The third case occurred in a newborn girl with congenital acute monocytic leukemia, leukemia cutis, and MCC [11]; and the last one was a pediatric hemodialysis patient who presented with metastatic brain calcifications and severe neurological manifestations secondary to uncontrolled hyperparathyroidism [12]. The clinical presentation of MCC is indistinct as cutaneous lesions might resemble xanthoma disseminatum and diffuse plane xanthomatosis [13], or common conditions such as molluscum contagiosum as in the present case.

In children with malignancy, hypercalcemia is uncommon. McKay and Furman reported that, over a period of 29 years, 25 out of 6,055 children treated for cancer were identified with hypercalcemia (0.4%) [1]. Kerdudo et al., over a period of 7 years, found 16 cases of hypercalcemia and reported a prevalence of 1.3% [14]. Recently, Moayeri et al. described a prevalence of hypercalcemia in 5.4% of patients, in which half of them were associated with acute lymphoblastic leukemia (ALL) [15].

There is no specific treatment; intense hydration, biphosphonates, and corticosteroids to reduce calcium levels are usually indicated [13]. Skin lesions could be resolved following a good control of the calcium and phosphate levels after a varied period of time [16].

To the best of our knowledge, this case may represent the first pediatric patient with pre-B cell ALL and MCC, as no case with similar skin lesions has previously been informed in the literature. Its presentation highlights the importance of skin features in the diagnosis of a potentially severe hematologic disease. It was clear that an accelerated hypercalcemia in children with ALL can confuse the clinician due to nonspecific symptoms that may delay its diagnosis and treatment. Although cutaneous lesions represent a later sign of involvement, they are indistinguishable from other conditions such as xanthomas; thus, the histological analysis plays an important role for its recognition. In conclusion, although hypercalcemia with MCC is infrequent, it can be the initial manifestation of ALL.
